# Selective Catalytic Oxidation of Benzyl Alcohol to Benzaldehyde by Nitrates

**DOI:** 10.3389/fchem.2020.00151

**Published:** 2020-03-20

**Authors:** Shurui Xu, Jie Wu, Peng Huang, Chunwen Lao, Hanchao Lai, Yuxiong Wang, Zhenyu Wang, Guoyu Zhong, Xiaobo Fu, Feng Peng

**Affiliations:** ^1^Engineering Research Center of None-food Biomass Efficient Pyrolysis and Utilization Technology of Guangdong Higher Education Institutes, Dongguan University of Technology, Dongguan, China; ^2^Key Laboratory of Distributed Energy Systems of Guangdong Province, School of Chemical Engineering and Energy Technology, Dongguan University of Technology, Dongguan, China; ^3^Guangzhou Higher Education Mega Center, School of Chemistry and Chemical Engineering, Guangzhou University, Guangzhou, China

**Keywords:** selective oxidation, benzyl alcohol oxidation, green oxidation, ferric nitrate, catalytic mechanism

## Abstract

In this paper, ferric nitrate was used to oxidize benzyl alcohol in a mild condition and demonstrated its better performance compared to HNO_3_. In the reaction, the conversion rate and product selectivity could be both as high as 95% in N_2_ atmosphere, while the benzaldehyde yield also reached 85% in air. Similar to Fe(NO_3_)_3_·9H_2_O, the other metallic nitrates such as Al(NO_3_)_3_·9H_2_O and Cu(NO_3_)_2_·3H_2_O could also oxidize the benzyl alcohol with high activity. The applicability of Fe(NO_3_)_3_·9H_2_O for other benzylic alcohol was also investigated, and the reaction condition was optimized at the same time. The results showed the Fe(NO_3_)_3_·9H_2_O would be more conducive in oxidizing benzyl alcohol under the anaerobic condition. The experiments in N_2_ or O_2_ atmospheres were conducted separately to study the catalytic mechanism of Fe(NO_3_)_3_. The results showed the co-existence of Fe^3+^ and ${\text{NO}}_{3}^{-}$ will generate high activity, while either was with negligible oxidation property. The cyclic transformation of Fe^3+^ and Fe^2+^ provided the catalytic action to the benzyl alcohol oxidation. The role of ${\text{NO}}_{3}^{-}$ was also an oxidant, by providing HNO_2_ in anaerobic condition, while ${\text{NO}}_{3}^{-}$ would be regenerated from NO in aerobic condition. O_2_ did not oxidize the benzyl alcohol conversion directly, while it could still be beneficial to the procedure by eliminating the unwelcome NO and simultaneously reinforcing the circulation of Fe^2+^ and Fe^3+^, which therefore forms a green cyclic oxidation. Hence, the benzyl alcohol oxidation was suggested in an air atmosphere for efficiency and the need of green synthesis.

## Introduction

Benzaldehyde (BzH) is one of the most important chemicals among the aromatic aldehyde family. It is used as the raw material for a large number of products, including perfume, beverage, pharmaceutical intermediates, and so on (Jachuck et al., 2006; Ragupathi et al., 2015; Ndolomingo and Meijboom, 2017; Zhu et al., 2017). Traditionally, BzH was synthesized by hydrolysis of benzal chloride or vapor/liquid-phase oxidation of toluene. In the former method, the chlorinated by-products and corresponding toxic acidic would be generated, which brought troubles to the industrial application (Mal et al., 2018; Lu et al., 2019), while the vapor/liquid oxidation of toluene was also limited because of the harsh reaction conditions and low selectivity (Miao et al., 2016). Recently, BzH production with benzyl alcohol oxidation was widely adopted in industry, based on its advantages of easy-control condition and high yield (Lv et al., 2018; Thao et al., 2018). In this method, potassium permanganate (KMnO_4_) (Mahmood et al., 1999) and dichromate (K_2_Cr_2_O_7_) (Thottathil et al., 1986) with a strong oxidizing property are chosen as oxidants. While those oxidants were not perfect industrial reagents, leading to a series of environmental issues and high cost.

Nitric acid (HNO_3_), as a rather inexpensive and high-performance oxidant, is commonly used in industry (Joshi et al., 2005; Aellig et al., 2012). For example, the niacin (vitamin B3) is synthesized from substituted pyridines oxidized by HNO_3_ (Yu et al., 2011). HNO_3_ is also regarded as the initiator in benzyl alcohol oxidation in the presence of O_2_ (Miao et al., 2011; Luo et al., 2012, 2014). In this approach, HNO_3_ initiates the oxidation of alcohols by decomposing NO_2_, which further formed HNO_2_ with H_2_O. HNO_2_ subsequently attacks substrate and generates the products by a series of reactions with releasing NO_x_. Finally, the HNO_3_ is regenerated by the NO_x_ oxidation by O_2_. However, the disadvantages of HNO_3_ should not be ignored totally, due to the risks of pollution and corrosion. Green oxidants such as hydrogen peroxide (H_2_O_2_) (Cánepa et al., 2017) and O_2_ (Yu et al., 2011; Cao et al., 2013, 2015; Zhu et al., 2017; Chen et al., 2018a,b; Yuan et al., 2018) have been attracting extensive attention for many years. It is noted that O_2_ or H_2_O_2_ itself has almost no activity and its oxidative performance needs to be activated by other materials.

Besides oxidants, the catalytic systems, including homogeneous and heterogeneous, have been developed. In the past decade, the heterogeneous precious metals catalysts, like Au (Zhan et al., 2012; Albadi et al., 2014), Pt (Liu et al., 2017), Ru (Ganesamoorthy et al., 2013), and Pd (Villa et al., 2010), were employed for selective oxidation of benzyl alcohols to BzH, based on their excellent performances. However, the high cost and limited resource of noble metals hindered their practical application. Moreover, the catalytic activity of heterogeneous catalysts would be lower than their homogeneous counterparts after several recycles (Parmeggiani and Camilla, 2012). Hence, the metal-based homogeneous catalysts, including Ru (Shimizu et al., 2005), V (Hanson et al., 2008), Cu (Hansen et al., 2013; Jia et al., 2014), and Fe (Jiang et al., 2016; Li et al., 2016; Miao et al., 2016; Hu et al., 2018), continued to gain great interests.

Among those homogenous catalysts, non-toxic, abundant, and bio-friendly, iron-based metals have widely aroused attentions (Martin and Suárez, 2002; Wang et al., 2005; Zhang et al., 2013; Hu et al., 2016, 2018). Zhang and co-workers reported a series of Fe catalysts, such as FeBr_3_, Fe_2_O_3_, and Fe_2_(SO_4_)_3_, with performance catalyzing benzylamine to imine in air atmosphere (Zhang et al., 2013). Wang et al. (2005) found an effective FeCl_3_/TEMPO/NaNO_2_ catalyst for benzyl alcohol oxidation, whose yield of benzaldehyde was as high as 99.0%. In this catalytic system, NaNO_2_ activated the catalytic reaction by releasing NO_2_, which oxidized Fe^2+^-TEMPOH to Fe^3+^-TEMPO. Consequently, Fe^3+^-TEMPO oxidized the benzyl alcohol to be benzaldehyde (Wang et al., 2005). Zhang and co-workers introduced Fe into imidazolium and successfully prepared a nice iron-based catalyst [Imim-TEMPO][FeCl_4_]/NaNO_2_ (Miao et al., 2011), showing a good activity for the selective oxidation of aromatic alcohols under 5% NaNO_2_. The catalytic mechanism of [Imim-TEMPO][FeCl_4_]/NaNO_2_ was similar to that of FeCl_3_/TEMPO/NaNO_2_, and the aromatic alcohol was oxidized in the process of redox between Fe^3+^ and NO_2_. Martin and co-workers (Martin and Suárez, 2002) discovered an efficient system by combining Fe(NO_3_)_3_ and FeBr_3_ for selective oxidation of benzylic alcohol to corresponding aldehyde. Though the Fe-based catalysts had good activity, problems are obvious. For example, the introduction of Br or co-catalyst (TEMPO) has increased the cost as well as environmental concerns, resulting in weaker competitive when industrial applications.

The reported Fe-based catalytic systems usually contain ${\text{NO}}_{2}^{-}$. In fact, ${\text{NO}}_{2}^{-}$ releases active NO_2_ and initiate the alcohol oxidation by several redox reactions. Inspired by this, we are intrigued whether the combination of metal iron (M^n+^) and ${\text{NO}}_{3}^{-}$/${\text{NO}}_{2}^{-}$ can construct an efficient catalytic system for the alcohol oxidation. Studies from Jachuck et al. (2006) and Dressen et al. (2009) verified that Fe(NO_3_)_3_ could successfully oxidize benzyl alcohol to benzaldehyde under microwave irradiation. They deemed Fe^3+^ in the oxidation of benzyl alcohol as catalyst, because overall Fe^3+^ remained in its original oxidative state. However, this study was limited in microwave irradiation and lacked the universality for actual application. Besides, the oxidation performances of other system composed of metal iron (Cu, Al, Mg, Co, Ni) and ${\text{NO}}_{3}^{-}$ /${\text{NO}}_{2}^{-}$ were unclear, and the role of M^n+^ had not been reported.

Hence, we studied the performance of Fe(NO_3_)_3_·9H_2_O by continuous heating in O_2_ and N_2_ (He) atmosphere. Interestingly, the results were different from the phenomenon in microwave irradiation. The catalytic performance of Fe(NO_3_)_3_·9H_2_O in N_2_ was significantly improved compared to that in O_2_. Further, the oxidation performance of other nitrates was studied. To expand the applicability of the combination of M^n+^ and ${\text{NO}}_{3}^{-}$, we conducted systematic research using Fe(NO_3_)_3_9H_2_O as an example. The reaction conditions such as temperature and solvent were optimized. The optimized conditions were applied to the oxidation of a variety of alcohols. Finally, the catalytic mechanism was put forward.

## Experimental

### Materials

Benzyl alcohol, nitrates with crystalline waters, and other reagents were analytical grade. Gas chromatography (GC) analysis was performed on SHIMAZDU GC-2014 equipped with a HP-5 column (30 m × 0.32 mm × 0.25 um) and a flame ionization detector.

### The Oxidation of Benzyl Alcohol

Typically, 3 mmol of benzyl alcohol, 3 mmol of naphthalene as an internal standard, and 15 mL of 1,4-dioxane as solvent were added to a 25 mL three-necked flask. Then the three-necked flask provided with a reflux condenser was evacuated using an aspirator and followed by the attachment of a nitrogen balloon. Subsequently, the solution was heated to the desired temperature. When the temperature of solution reached the set point, 2 mmol of ferric nitrate (Fe(NO_3_)_3_·9H_2_O) was added into it. Samples were taken at appropriate intervals through a silicon septum using a hypodermic needle and were filtered with a membrane filter (PVDF) with 13 mm × 0.22 μm pore prior to GC analysis.

### The Product Analysis

The analysis of benzyl alcohol and oxidation products was carried out on Aglient 7980 series with a HP-5 column and a flame ionization detector. The condition of GC for the HP-5 capillary column (30 m, DF = 0.25 mm, 0.25 mm i.d.), and temperature program was carried out (initial temperature = 100°C, 3 min; final temperature = 250°C, heating rate = 10°C min^−1^, temperature of injector = 280°C, temperature of detector = 280°C). The quantitative results of products were based on the internal standard method, using naphthalene as an internal standard. The typical analytic procedure was as following: (1) 0.4 ml sample was taken from reaction solutions and was then filtered with a membrane filter (PVDF) with 13 mm × 0.22 μm; (2) then, the sample would be diluted 10-fold before GC analysis; (3) when the GC temperature reached the set points, the diluted solution was injected into this equipment to analyze products according to the different retention times and response peak area. The typical results of GC measurements and internal standard working curve were shown in Figure S1. The results reported as conversion and selectivity are expressed in mol%, based on the total benzyl alcohol intake. The calculation of the conversion and selectivity was as follows:

(1) $$\begin{array}{l} \text{Conversion of benzyl alcohol}\\ =\frac{\text{Moles of benzyl alcohol reacted }}{\text{Initial moles of benzyl alcohol}}\times \text{100}\% \end{array}$$

(2) $$\begin{array}{l} \text{Selectivity of benzaldehyde}\\ =\frac{\text{Moles of benzaldehyde formed}}{\text{Moles of benzyl alcohol reacted}}\times \text{ 100}\% \end{array}$$

(3) Yield  of benzaldehyde= Conversion ×Selectivity

## Results and Discussion

### The Oxidation of Benzyl Alcohol by Ferric Nitrate

**Figure 1:**
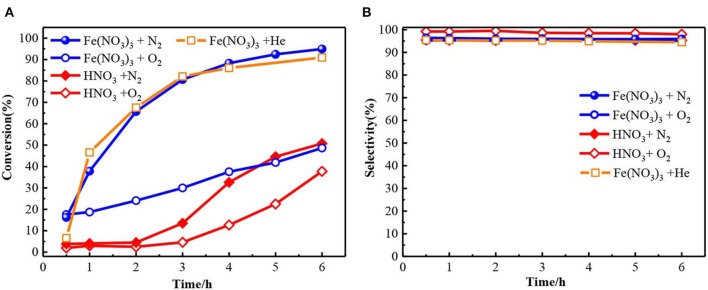
The conversion **(A)** and selectivity **(B)** for benzyl alcohol oxidation to benzaldehyde with Fe(NO_3_)_3_·9H_2_O and HNO_3_. Fe(NO_3_)_3_ denotes Fe(NO_3_)_3_·9H_2_O unless otherwise noted.

Figures 1A,B showed the oxidation results of benzyl alcohol to benzaldehyde in the presence of ferric nitrate and nitric acid. In N_2_ condition, the conversion of benzyl alcohol catalyzed by ferric nitrate was 94.9% after 6 h. To ensure reproducibility, the experimental error-based three parallel experiments was shown in Figure S2 and the carbon balance during experiments was also evaluated (Table S1). The results showed that performance of Fe(NO_3_)_3_ had a good testing repeatability and was highly efficient. Interestingly, the benzyl alcohol conversion catalyzed by Fe(NO_3_)_3_ could be as high as 96.84% with 94.5% selectivity when replacing N_2_ with He. Compared with HNO_3_, ferric nitrate exhibited an excellent activity in the benzyl alcohol oxidation. The conversion of benzyl alcohol catalyzed by ferric nitrate was 46.2%—higher than that by nitric acid. Similarly, under aerobic conditions (O_2_), the conversion of benzyl alcohol catalyzed by ferric nitrate was about 13% higher than that by nitric acid with similar selectivity. The results indicated the oxidation performance of ferric nitrate was markedly better than that of nitric acid, no matter under anaerobic or aerobic conditions.

Compared with other typical catalysts, the catalytic activity of Fe(NO_3_)_3_ was also outstanding. The conversion of benzyl alcohol catalyzed by Fe(NO_3_)_3_ with any oxidant (96.84%) was close to that by CNT-HNO_3_ (Luo et al., 2012) (96.2%), as shown in Table 1 (Entry 2). Besides, the performance of Fe(NO_3_)_3_ surpassed the typical metal-based catalyst, such as Au/γ-Al_2_O_3_ (73.4%) (Ndolomingo and Meijboom, 2017), Fe/MCM41 (55%) (Cánepa et al., 2017), or Fe-N-C (78.0%) (Xie et al., 2017). Moreover, Fe(NO_3_)_3_ also exhibited comparable activity with transition-metal oxides, such as Co_3_O_4_/RGO-_N_ (93.9%) (Nie et al., 2013), MnO_x_ (72.7%) (Jing et al., 2007). The excellent catalytic activity of Fe(NO_3_)_3_ may be attributed to the combined action of Fe^3+^ and ${\text{NO}}_{3}^{-}$. On the one hand, ${\text{NO}}_{3}^{-}$ could produce NO_2_, and then NO_2_ with H_2_O was converted to HNO_2_, which had been proved as a pivotal role in benzyl alcohol oxidation (Aellig et al., 2012; Luo et al., 2012). On the other hand, the valence change of Fe could catalyze benzyl alcohol oxidation according to the literatures (Miao et al., 2016; Hu et al., 2018). Coincidentally, the transformation between Fe^3+^ and Fe^2+^ was demonstrated by the K_3_[Fe(CN)_6_] solution. As shown in Figure S3, the Prussian blue precipitate appeared in the experimental process due to the existence of Fe^2+^. Therefore, Fe(NO_3_)_3_ exhibited the excellent oxidation activity via Fe^3+^ initiating a series of electron and proton transfer. It was noted that the anaerobic condition was beneficial to improve the oxidation performance of ferric nitric. The reason is discussed in detail in mechanism Part 3.4.

**Table 1:** Catalytic results for benzyl alcohol oxidation with different catalysts.

**Entry**	**Material**	**Oxidant**	**Temperature [^**°**^C]**	**Time [h]**	**Con. [%]**	**Sel. [%]**	**References**
1	Fe${\left({\text{NO}}_{\text{3}}\right)}_{3}^{\text{a}}$	–	80.0	6	96.84	94.5	This work
2	CNT+HNO_3_	O_2_	90.0	5	96.2	88.3	Luo et al., 2012
3	Au-γAl_2_O_3_	TBHP	125.0	5	73.4	84.4	Ndolomingo and Meijboom, 2017
4	Fe/MCM41	H_2_O_2_	70.0	7	55	90.0	Cánepa et al., 2017
5	NG-900	O_2_	70.0	3	12.8	100.0	Long et al., 2012
6	Fe-N-C	${\text{O}}_{2}^{\text{b}}$	80.0	8	78.0	90.0	Xie et al., 2017
7	Au/Al_2_O_3_	O_2_	130.0	5	69.0	65.0	Choudhary et al., 2005
8	CeO_2_	H_2_O_2_	50.0	6	68.0	92.0	Tamizhdurai et al., 2017
9	Co_3_O_4_	O_2_	100.0	7	38.6	67.6	Nie et al., 2013
10	Co_3_O_4_/RGO-_N_	O_2_	100.0	7	93.9	>99.0	Nie et al., 2013
11	MnO_x_	O_2_	80.0	3	72.7	–	Jing et al., 2007
12	NiO_2_	O_2_	90.0	6	80.0	100.0	Ji et al., 2005
13	CrBO_3_	O_2_	90.0	5	41	51	Öztürk et al., 2008
14	Co-ZIF-67	O_2_	100.0	8	50.0	97.6	Yang et al., 2016

*^a^ The benzyl alcohol oxidations were conducted in the inert atmosphere*.

*^b^ The pressure of O_2_ in this reference was 1 MPa*.

### The Performance of Other Metallic Nitrates

**Figure 2:**
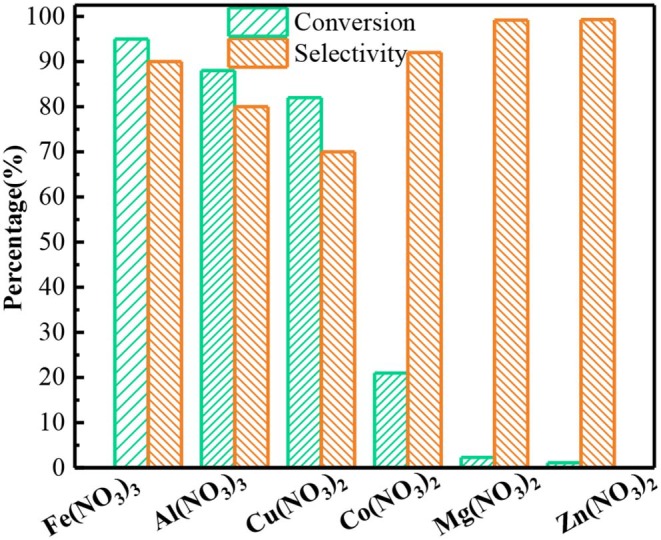
The oxidation of benzyl alcohol to benzaldehyde by different nitrates. Reaction conditions: benzyl alcohol (3 mmol), 1,4-dioxane (15 mL), Fe(NO_3_)_3_ and Al(NO_3_)_3_ (2 mmol), or other nitrates (3 mmol), 80°C, 6 h ball N_2_.

Subsequently, the catalytic performance of other nitrates was also studied. The benzyl alcohol oxidation catalyzed by other nitrates including was investigated, as shown in Figure 2. The result proves other nitrates are also capable for converting the benzyl alcohol to benzaldehyde. In Al(NO_3_)_3_ system, the conversion of benzyl alcohol and the selectivity to benzaldehyde was 88.1% and 80% after 6 h, lower than those in Fe(NO_3_)_3_ system. Similarly, Cu(NO_3_)_2_ could also oxidize the benzyl alcohol with the conversion of 82.3%. While the selectivity to benzadehyde was only 70%, and the other 30% was benzoic acid from the excessive oxidation. The catalytic performance of Co(NO_3_)_2_ significantly decreased. While Mg(NO_3_)_2_ and Zn(NO_3_)_2_ had almost no catalytic activity. The order of activity of different nitrates is Fe(NO_3_)_3_ > Al(NO_3_)_3_ > Cu(NO_3_)_2_ > Co(NO_3_)_2_ > Mg(NO_3_)_2_ ≈ Zn(NO_3_)_2_. Yuvaraj et al. (2003) tested the decomposition temperature of these nitrates by TG/DAT. And they found Fe(NO_3_)_3_ and Al(NO_3_)_3_ had the lowest the decomposition temperature (130°C), followed by Cu(NO_3_)_2_ (227°C) and Co(NO_3_)_2_ (247°C), and Zn(NO_3_)_2_ (367°C) was the highest. Their decomposition products were metal oxide, NO_2_ and O_2_. NO_2_ and H_2_O together would convert into HNO_2_ which could attack the benzyl alcohol, finally producing benzaldehyde. Hence, the nitrates with lower decomposition temperature could easily produce NO_2_ and oxidize more benzyl alcohol. As expected, the rank of conversion of benzyl alcohol in different nitrate system was related to the decomposition temperature of these nitrates.

### Effect of Reaction Conditions on Oxidation Performance of Fe(NO_3_)_3_

**Figure 3:**
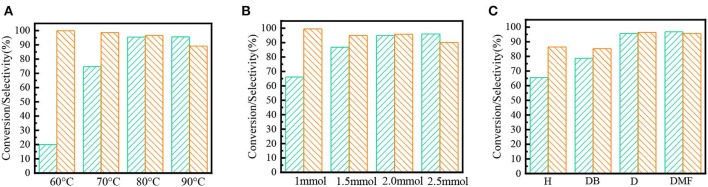
The effect of different factors on the oxidation activity of Fe(NO_3_)_3_. Green square: conversion; Orange square: selectivity; **(A)** temperature; **(B)** dosage of Fe(NO_3_)_3_; **(C)** solvent polarity: n-hexane (H), dimethylbenzene (DB); 1,4-dioxane (D); N,N-dimethylformamide (DMF).

Fe(NO_3_)_3_ is an excellent catalyst among several nitrates; the effect of reaction condition and solvent on the performance of Fe(NO_3_)_3_ was investigated. As the results shown in Figure 3A, the higher temperature would markedly enhance the yields of benzaldehyde. Since high temperature would increase the risk of the over oxidation from benzaldehyde to benzoic acid, the optimum temperature was 80°C. Similarly, 2 mmol Fe(NO_3_)_3_ could selectively oxidize the benzyl alcohol to benzaldehyde with 91.5% yields shown in Figure 3B. Besides, the solvent had an important effect on the activity of Fe(NO_3_)_3_. The results in Figure 3C show the strong polar solvent could bring a mutual solution containing the aqueous (HNO_2_) and organic phase (benzyl alcohol), which would be more favorable to form of benzyl nitrite, resulting in high conversion and selectivity.

### The Applicability of Fe(NO_3_)_3_ Catalyst

Subsequently, to demonstrate the general applicability of Fe(NO_3_)_3_, selective oxidation of substituted benzyl alcohols with different functional groups was investigated. The results presented in Table 2 showed all these primary benzylic alcohols could be converted to corresponding aldehydes. The oxidation results were somewhat related to the substituent groups on the phenyl ring. The alcohols with electron withdrawing groups (–NO_2_, –Cl) gained lower yields of products than those with electron donating groups (–MeO, –OH). The effect rule of substituent groups in Fe(NO_3_)_3_ system is consistent with that in HNO_3_ system (Joshi et al., 2005). The formation of benzyl nitrite, a vital intermediate product, can be regarded as the electrophilic substitution reaction of benzyl alcohol. Thus, the electron-donating substituents would enhance the yield of product by increasing the electron density on the benzyl ring and vice versa. The results proved that the electron density on the aromatic ring played a critical role in the oxidation of benzylic alcohol.

**Table 2:** The oxidation of different alcohols by Fe (NO_3_)_3_.

**Samples**	**Conversion**	**Selectivity**	**Yield**
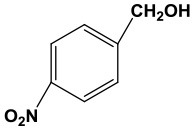	72.3	90.1	65.1
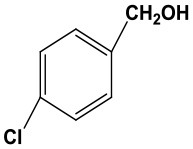	82.6	91.0	75.1
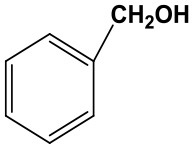	94.9	94.8	89.7
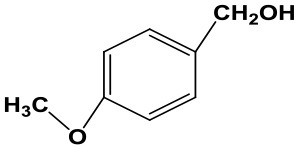	96.8	95.0	91.9
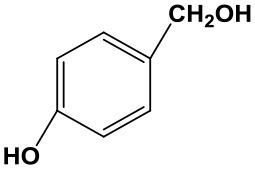	98.2	95.1	93.4

*Reaction conditions: Alcohol (3 mmol); 1,4-dioxane (15 mL); Fe(NO_3_)_3_ (2 mmol); 80°C, 6 h ball N_2_*.

### Reaction Mechanism

Combined with the experimental results and literature reports, the following catalytic mechanism hypothesis was preliminarily put. Both Fe^3+^ and ${\text{NO}}_{3}^{-}$ may have an important effect on the oxidation process. Fe^3+^ provides catalytic function by the electron transferring, while the NO_x_ produced from the experimental process may have certain oxidation performance. To verify above possible speculations and analyze the catalytic mechanism, a series of experiments were designed, and the results were shown as follows.

**Figure 4:**
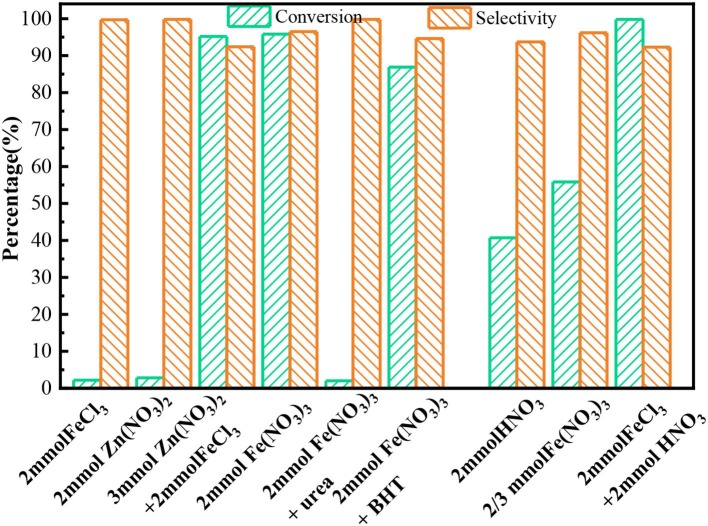
The conversion and selectivity for the oxidation of benzyl alcohol in different system. Benzyl alcohol (3 mmol), 1,4-dioxane (15 mL), 80°C, 6 h, ball N_2_.

To illustrate the role of Fe^3+^ and ${\text{NO}}_{3}^{-}$, Zn(NO_3_)_2_ or FeCl_3_ was individually added to the reaction mixtures. The result showed benzyl alcohol conversion in both systems was very low (Figure 4), indicating that only Fe^3+^ or ${\text{NO}}_{3}^{-}$ had poor oxidation activity. While in Zn(NO_3_)_2_ + FeCl_3_ system, the conversion of benzyl alcohol was as high as 95.8% with 93.6% selectivity, which was similar to that in Fe(NO_3_)_3_ system with the same amount of Fe^3+^ and ${\text{NO}}_{3}^{-}$. The results proved the Fe^3+^ and ${\text{NO}}_{3}^{-}$ together could oxidize the benzyl alcohol. To illuminate how Fe^3+^ and ${\text{NO}}_{3}^{-}$ together catalyze the benzyl alcohol, introducing urea as a known HNO_2_ scavenger into Fe(NO_3_)_3_ system. The results in Figure 4 showed the catalytic activity of Fe(NO_3_)_3_ was almost prevented. This phenomenon proved that the oxidation process of Fe(NO_3_)_3_ was mainly achieved by HNO_2_ attacking benzyl alcohol. The intermediate benzyl nitrite detected by GC-MS in our experiments further verified the reaction between benzyl alcohol and HNO_2_. Besides, when butylated hydroxytoluene (BHT) as a radical trapping agent was added into the Fe(NO_3_)_3_ system, the conversion still kept a high level of 86.3%, proving that the benzyl alcohol oxidation catalyzed by Fe(NO_3_)_3_ is not a radical-involved reaction. Interestingly, the conversion would increase obviously, and the selectivity also kept at a high value (95%) when Fe^3+^ instead of H^+^ with the same mole of ${\text{NO}}_{3}^{-}$ as shown in Figure 4. Furthermore, the addition of Fe^3+^ into HNO_3_ system also significantly enhanced the conversion of benzyl alcohol compared with the HNO_3_ system. From these results, we speculated Fe^3+^ had a special catalytic function in the process of benzyl alcohol oxidation.

**Figure 5:**
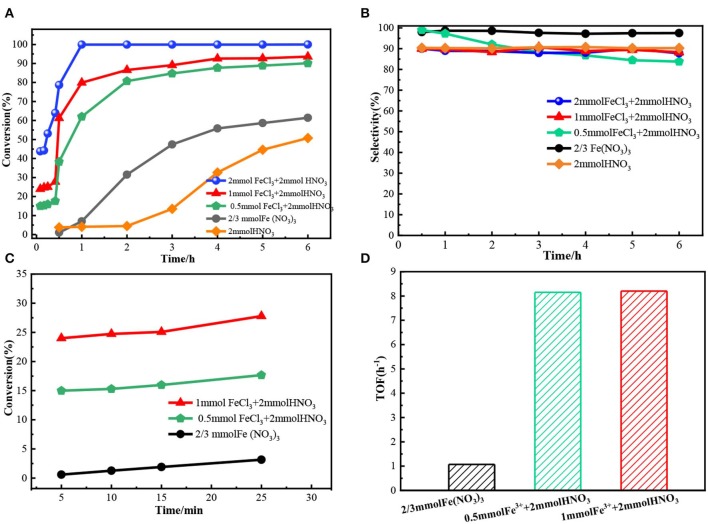
The oxidation results of benzyl alcohol in different catalyst systems. The TOF was defined as $\frac{benzyl\;alcohol\;converted\left(g\right)}{Fe\;content\left(g\right)\;\times reaction\;time}$ and were calculated at 15 min, for which the conversion was lower than 25.0%. **(A)** The benzyl alcohol conversion in 6 h. **(B)** The selectivity of benzaldehyde. **(C)** The benzyl alcohol conversion in 30 min. **(D)** TOF.

A series of experiments were conducted to further illuminate the catalysis of Fe^3+^. As the results shown in Figure 5A, the reaction rate increased as the amount of Fe^3+^ increased in FeCl_3_-HNO_3_ system. Meanwhile, the results shown in Figure 5B proved that the variation of selectivity was little. To quantify the effect of Fe^3+^, the turnover frequency (TOF) was calculated based on Fe content at the conversion lower than 25.0%, as shown in Figure 5C. The results proved the Fe^3+^ had a remarkable effect on the oxidation reaction. The TOF of Fe^3+^ in the FeCl_3_-HNO_3_ system further enlarged compared to that in the Fe(NO_3_)_3_ system. As shown in Figure 5D, the TOF of Fe^3+^ in the FeCl_3_-HNO_3_ system was almost unchanged (8.1 h^−1^), even if the amount of Fe^3+^ increased from 0.5 to 1 mmol.

**Figure 6:**
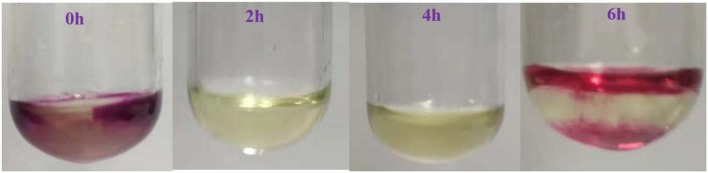
The phenomena in the experimental process at different moments after adding 1 mol/L KMnO_4_ to the reaction solution.

Furthermore, the change of Fe in benzyl alcohol oxidation was analyzed by experiments. The transformation between Fe^3+^ and Fe^2+^ was detected *in situ* by 1 mol/L KMnO_4_ solution in the oxidation process. For comparison, the reaction solvent (1,4-dioxane) was added to the KMnO_4_ solution, the solution was black-purple as shown in Figure 6. The color of KMnO_4_ solution changed from black-purple to yellow when KMnO_4_ solution was added into reaction solution at 2 h. This phenomenon showed the KMnO_4_ was reduced by Fe^2+^, and the Fe^2+^ existed in the reaction process. However, the KMnO_4_ solution changed from yellow to brown when it was added into the reaction solution at the reaction time of 6 h. The phenomenon illustrated that there were less Fe^2+^ in the reaction solution. Because the oxidation reaction of benzyl alcohol almost completed, most of Fe^3+^ did not convert anymore and Fe^2+^ would further oxidized to Fe^3+^ by the O_2_ from the decomposition of Fe(NO_3_)_3_. So, the cyclic conversion of Fe^3+^ and Fe^2+^ really occurred in benzyl alcohol oxidation. According to the reported results, the valence transformation of metal iron such as Mn^III^/Mn^II^ (Yang et al., 2014; Fei et al., 2017; Gurrala et al., 2018), Co^III^/Co^II^ (Zhou et al., 2015; Cordoba et al., 2017; Li et al., 2017), Cr^III^/ Cr^II^ (Thao et al., 2018) could catalyze the oxidation of alcohols. Especially, the transformation of Fe^3+^/Fe^2+^ possessed high catalytic activity by initiating a series of electron transfer (Hu et al., 2016, 2018; Miao et al., 2016). Combined with above results, Fe^3+^ could be regard as a catalyst and the electron transfer between of Fe^3+^ and Fe^2+^ would catalyze the oxidation process.

**Figure 7:**
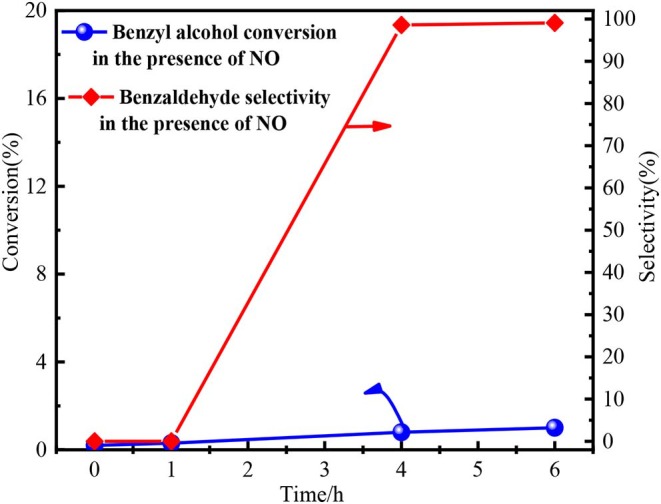
The performance of NO in benzyl alcohol oxidation process. Reaction condition: 3 mmol benzyl alcohol, 15.0 ml 1,4-dioxane, 80°C.

Finally, the function of NO_x_ was investigated by a series of auxiliary experiments. As we know, nitric oxide (NO) would be immediately oxidized to nitrogen dioxide (NO_2_) by O_2_ (Wang et al., 2005; Shen et al., 2015, 2019; Miao et al., 2016; Hu et al., 2018; Dong et al., 2019; Zhao et al., 2019). O_2_ was flowed into the reaction system in the experimental process to detect the NO. Brown fumes immediately occurred (Figure S4), proving the presence of NO in the inert atmosphere. Subsequently, NO was prepared by the reaction between Cu and 35 wt % nitric acid (3Cu + 8HNO_3_ → 3Cu(NO_3_)_2_ + 2NO + 4H_2_O). The oxidation of benzyl alcohol by NO was conducted and the detailed experimental process was shown in Figure S5. The conversion of benzyl alcohol by NO was only 1% at 6 h with 99% selectivity, as shown in Figure 7. The results indicated NO itself could not oxidize benzyl alcohol. Hence, the NO_2_ from the decomposition of Fe(NO_3_)_3_ played the key role in oxidation process by forming HNO_2_.

**Scheme 1:**
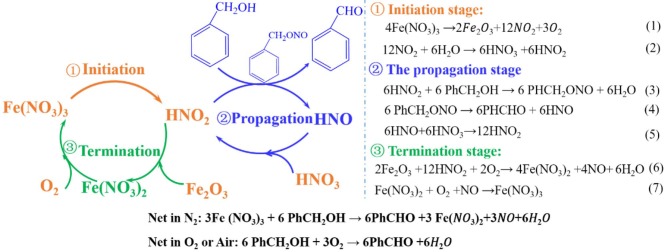
The probable routes for oxidation of benzyl alcohol by Fe(NO_3_)_3_.

According to the above investigation, the mechanism of the benzyl alcohol oxidation in N_2_ condition was put forward and shown in Scheme 1. At the suitable reaction temperature, the Fe(NO_3_)_3_ decomposes into Fe_2_O_3_, NO_2_, and O_2_ (Yuvaraj et al., 2003) by Equation 1. Subsequently, the HNO_2_ and HNO_3_ can be formed by the reaction between NO_2_ and H_2_O, see Equation 2. Then, the oxidation reaction successfully gets into the propagation stage and HNO_2_ attacks the benzyl alcohol (PhCH_2_OH) and gives benzyl nitrite (PhCH_2_ONO). Benzyl nitrite decomposes into benzaldehyde (PhCHO) and HNO at experimental temperature by Equation 4. In the termination stage, as shown in Equation 6, Fe(NO_3_)_2_ can be formed by the reaction between the HNO_2_ and Fe_2_O_3_ with consuming O_2_. At the same time, Fe(NO_3_)_2_ is oxidized to Fe(NO_3_)_3_ by O_2_. Consequently, from Reaction 1 to Reaction 7, ${\text{NO}}_{3}^{-}$ can be considered as the real oxidant, and the Fe ion acts a catalyst through the transformation between Fe^2+^ and Fe^3+^.

**Figure 8:**
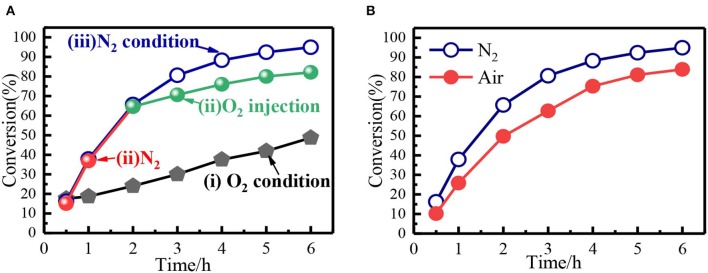
**(A)** The benzyl alcohol conversion under different conditions: (i) O_2_; (ii) N_2_ (1-2 h) and O_2_ (2–6 h); (iii) N_2_. **(B)** Comparison of the benzyl alcohol conversion in N_2_ and air.

From the reaction mechanism, when the system was filled with O_2_, the decomposition of Fe(NO_3_)_3_ would be hindered, leading to low conversion compared with anaerobic condition (N_2_ or He). Interestingly, the O_2_ was double-edged gas since it could oxidize the Fe^2+^ to Fe^3+^ and eliminate NO Equation 7. In the presence of adequate oxygen, the Fe^2+^ was completely oxidized into Fe^3+^, and oxidative state of Fe remained the same before and after the reaction. The conversion of benzyl alcohol in Fe(NO_3_)_3_-O_2_ system was relatively low (Figure 8A), but it was still higher than that in HNO_3_ system. Moreover, in Fe(NO_3_)_3_ system, the conversion of benzyl alcohol could reach 82% when O_2_ replaced N_2_ after the reaction continuing 2 h (Figure 8A). As expected, the conversion of benzyl alcohol in air (N_2_ + O_2_) condition was close to that in N_2_ condition (Figure 8B). In the aerobic conditions, ${\text{NO}}_{3}^{-}$ would be regenerated and the Fe^2+^ was completely oxidized to Fe^3+^, indicating O_2_ was the actual oxidation. Though the O_2_ would be not good for the high conversion, it could build a green cyclic oxidation process via removing NO and regenerating Fe(NO_3_)_3_. Hence, the benzyl alcohol conversion would be relatively high in air atmosphere, which also meet the need of green synthesis due to removing the NO.

## Conclusion

As a bio-friendly and economical material, ferric nitrate showed an outstanding oxidation performance for benzyl alcohol oxidation. The conversion of benzyl alcohol in ferric nitrate system reached 95%, which was 46% higher than that in nitric acid system under N_2_ atmosphere. Other metallic nitrates that could release NO_2_ at reaction temperature also had high properties for benzyl alcohol oxidation. Moreover, ferric nitrate is of excellent applicability for other primary benzylic alcohols oxidation under optimized condition. The mechanism study indicated ferric nitrate was as initiator in the reaction. In the procedure, it would decompose into Fe_2_O_3_ and NO_2_ which immediately became HNO_2_, attacking benzyl alcohol, and forming the benzaldehyde afterward. While in anaerobic atmosphere, ${\text{NO}}_{3}^{-}$ was the oxidant by providing HNO_2_ and the transformation cycle between Fe^3+^ and Fe^2+^ generates a catalytic effectiveness. Hence, the balance of high conversion and green synthesis requirement would be obtained for the benzyl alcohol oxidation in the air atmosphere.

## Data Availability Statement

The datasets generated for this study are available on request to the corresponding author.

## Author Contributions

SX, GZ, and FP designed experiments. JW, PH, CL, HL, YW, and ZW carried out experiments. SX, GZ, XF, and FP analyzed experimental results, analyzed data, and wrote the manuscript.

### Conflict of Interest

The authors declare that the research was conducted in the absence of any commercial or financial relationships that could be construed as a potential conflict of interest.
