# Supplementary material for: Selective Catalytic Oxidation of Benzyl Alcohol to Benzaldehyde by Nitrates
Source: Front Chem. 2020 Mar 20;8:151. doi: 10.3389/fchem.2020.00151 (PMC7099050; doi:10.3389/fchem.2020.00151)
Supplement: Supplementary file 1 [file Data_Sheet_1.pdf]

Supplementary Material

Table of Contents

1. The GC measurement and the calculation analysis of products
2. The error bar and balance of Carbon
3. The analysis of Fe^{2+} in alcohol oxidation reaction
4. The analysis of off-gas in alcohol oxidation reaction
5. The benzyl alcohol oxidation by NO

1. The GC measurement and the calculation analysis of products

(I): Sample analysis was made on Aglient 7980 series with a HP-5 column. As the Figure S1(a) shown, the products and substrate could be well separated and the retentions time of benzyl alcohol, benzaldehyde and benzoic acid and internal standard were 4.663min, 6.638min, 10.99min and 11.757min.

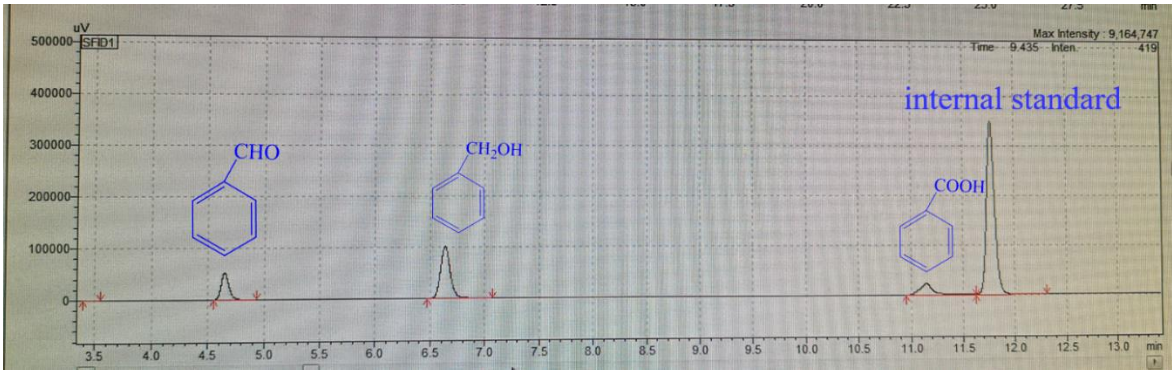

Figure S1 (a). Typical GC diagram of benzyl alcohol oxidation to benzaldehyde.

(II) : The quantitative analysis of different products was based on internal standard method. The relationship between peak area ration and mole ration of product to internal standard was obtained using linear regression method. The standard curves of benzyl alcohol, benzaldehhde and benzoic acid with 0.9998 goodness of fit were shown as follows.

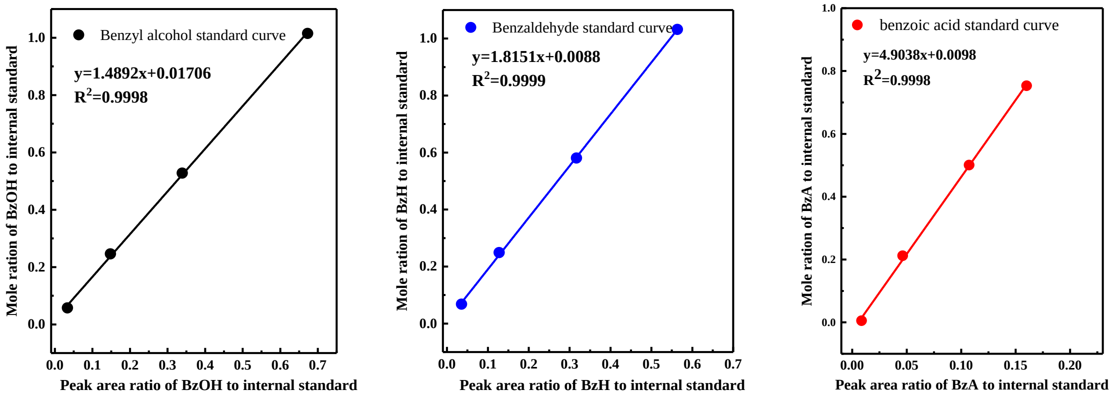

Figure S1(b). The standard curves of benzyl alcohol, benzaldehyde and benzoic acid.

2. The error bar and balance of Carbon

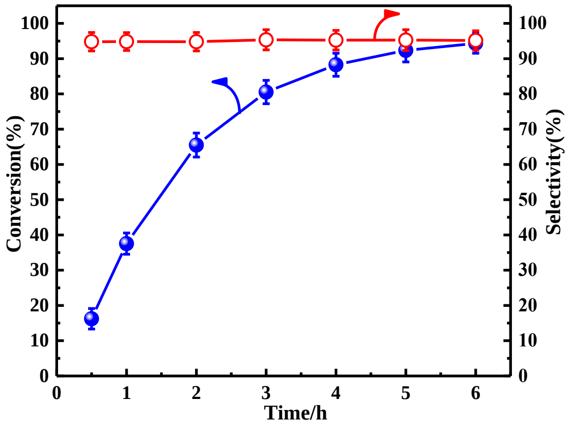

-

Figure S2 The average results for benzyl alcohol oxidation to benzaldehyde in the presence of $Fe(NO_3)_3$. The error bars were based on three experiments.

Table S1 The product distribution and carbon balance in the process of benzyl alcohol oxidation with $Fe(NO_3)_3$ at different time.

Time[h]	Amount of substrate(mmol)	Con.[%]	Amount of product(mmol)		Carbon balance[%]
	BzOH		BzH	BzA	
0	3.000	0	0	0	100.00%
0.5	2.5141	16.19	0.4651	0.0249	100.13%
1	1.8648	37.84	1.078	0.0567	99.96
2	1.0293	65.69	1.8720	0.0982	99.98
3	0.5811	80.63	2.2974	0.1206	99.97
4	0.3516	88.28	2.5159	0.1324	99.99
5	0.2274	92.42	2.6339	0.1388	100.00%
6	0.1521	94.93	2.6899	0.1526	99.82%

Reaction conditions: Benzyl alcohol (3 mmol), 1,4-dioxane (15 mL), 6 h, ball N_2, 80°C.

3 The analysis of Fe^{2+} in alcohol oxidation reaction

In order to prove the presence of the key intermediate, a series of experiments was carried out. Firstly, the Fe^{2+} was detected by $K_3[Fe(CN)_6]$ solution. As we know, the $Fe_3[Fe(CN)_6]_2$ which was blue precipitates would be formed when $K_3[Fe(CN)_6]$ was added to the Fe^{2+} solution. After 4 h of benzyl alcohol oxidation reaction, two kinds of solution from aerobic (ball O_2) and anaerobic (ball N_2) condition were obtained. At the same time, the 1mol/L $K_3[Fe(CN)_6]$ solution was added to above reaction solution. The pictures A and B in Figure S3 represent the results in O_2 condition and N_2 condition, respectively. For the reaction solution in O_2 condition, there was no blue precipitates when $K_3[Fe(CN)_6]$ was added, which proved there was no Fe^{2+} in the solution at the end of reaction. Contrary, there was Fe^{2+} in solution under N_2 condition. It proved that Fe^{2+} existed in solution as the form of $Fe(NO_3)_2$ at the end of reaction in N_2 condition.

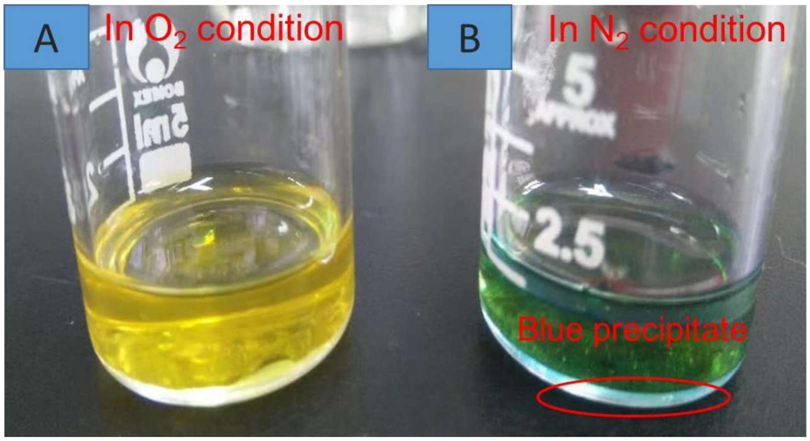

Figure S3. The result at (A) O_2 and (B) N_2 condition.

4. The analysis of off-gas in alcohol oxidation reaction

Further, the experiment was designed to test whether there were nitrogen oxides at the end of oxidation reaction. Figure S4(a) shows the off-gas of oxidation reaction in the condenser pipe was colorless. While the colorless gas immediately turned into brown fume when the O_2 flowed into the condenser pipe. And the brown fume was attributed to the formation of NO_2, which was attributed to the reaction between NO and O_2. So there was NO at the end of the oxidation reaction in N_2 condition.

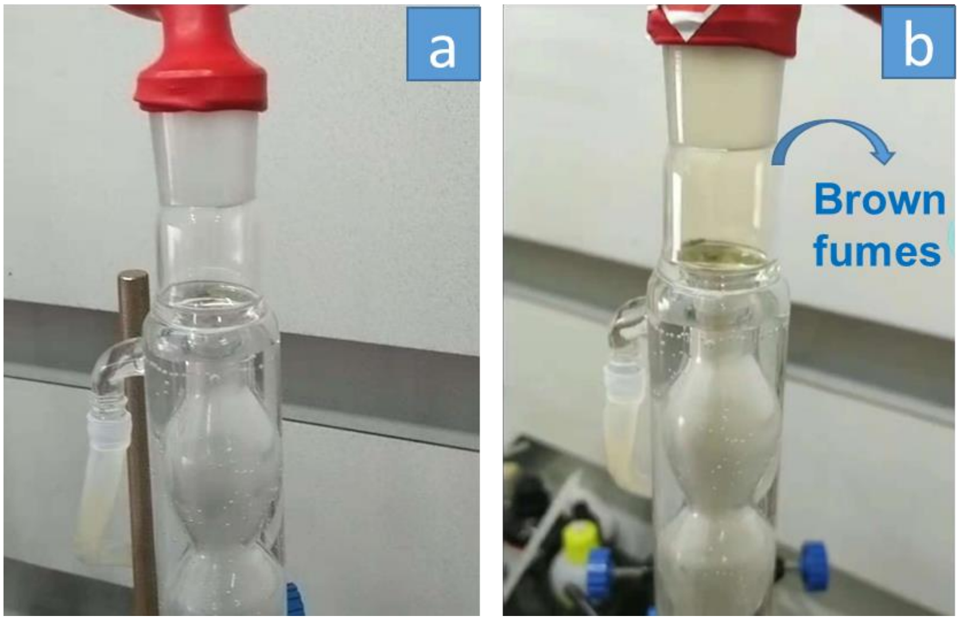

Figure S4. The color of gas before (a) and after (b) of the condenser pipe flowed into O_2.

5 The benzyl alcohol oxidation by NO

As shown in Figure S5(a), the NO was prepared by Cu and 35wt% dilute nitric acid solution in protective nitrogen ($3Cu+8HNO_3 \rightarrow 3Cu(NO_3)_2+2NO+4H_2O$). At the same time, the prepared NO would be put into benzyl alcohol solution in the atmosphere of nitrogen (Figure S6(b)).

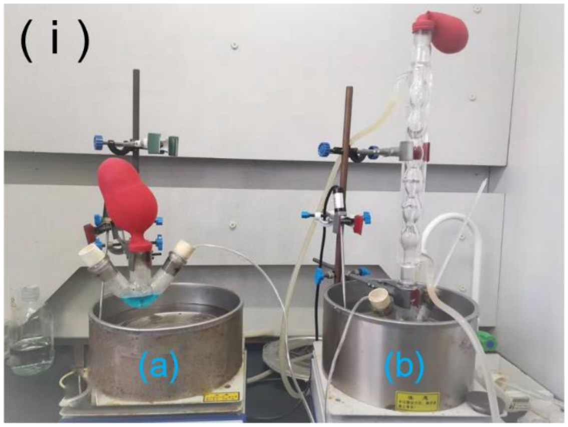

Figure S5. (a) The preparation of NO by Cu and HNO_3 at 25 °C in protective nitrogen; (b) the benzyl alcohol oxidation in NO atmosphere. Reaction condition: 3 mmol benzyl alcohol, 15ml 1,4-dioxane, 80°C.
